# Convalescent plasma for COVID-19: male gender, older age and hospitalisation associated with high neutralising antibody levels, England, 22 April to 12 May 2020

**DOI:** 10.2807/1560-7917.ES.2020.25.45.2001754

**Published:** 2020-11-12

**Authors:** Jennifer Mehew, Rachel Johnson, David Roberts, Heli Harvala

**Affiliations:** 1 Statistics and Clinical Studies, NHS Blood and Transplant, Bristol, United Kingdom; 2 NHS Blood and Transplant, Oxford, United Kingdom; 3 Radcliffe Department of Medicine and BRC Haematology Theme, University of Oxford, John Radcliffe Hospital, Oxford, United Kingdom; 4 Microbiology Services, NHS Blood and Transplant, London, United Kingdom

**Keywords:** SARS-CoV-2, COVID-19, convalescent plasma, neutralising antibody levels, multivariable model, plasma donor

## Abstract

We analysed factors associated with neutralising antibody levels in 330 convalescent plasma donors. Women and younger donors were more likely not to have measurable neutralising antibodies, while higher antibody levels were observed in men, in older donors and in those who had been hospitalised. These data will be of value in the timely recruitment of convalescent plasma donors most likely to have high levels of neutralising antibodies for ongoing studies investigating its effectiveness.

At the time of a second wave of the coronavirus disease (COVID-19) pandemic caused by severe acute respiratory syndrome coronavirus 2 (SARS-CoV-2) [1], effective antiviral therapies and vaccines are not yet available for clinical use [2,3]. Convalescent plasma collected from recovered COVID-19 patients has been suggested a safe and probably effective treatment in some non-randomised studies [4-11]. As the efficacy of such therapy is most probably associated with the presence of high levels of neutralising antibody in the donated plasma, we investigated the clinical and demographic factors that are predictive of high titres in convalescent plasma donors. This may provide an effective and rapid way to support convalescent plasma collections for ongoing randomised clinical trials.

## Collection of convalescent plasma

We collected convalescent plasma via apheresis from individuals with suspected (self-reported COVID-19 symptoms) or laboratory-confirmed (PCR) SARS-CoV-2 infection at least 28 days after the symptom resolution in England between 22 April and 12 May 2020, using otherwise the standard donor selection guidelines in the United Kingdom (UK). Donor recruitment was enhanced via social and paper media campaigns. We collected a total of 436 donations from donors aged between 17 and 65 years during the study period and tested all these donations for neutralising antibodies against SARS-CoV-2 as previously described [12,13]. From these, we included in this study 330 donors who had a previous PCR-confirmed SARS-CoV-2 infection and had detectable antibodies against SARS-CoV-2.

## Characteristics of convalescent plasma donors

We extracted donor characteristics data from the NHS Blood and Transplant donor management database (Supplementary Table S1). Most donors were male (216/330, 65%), with white ethnic background (224/330, 68%) and had blood group A (149/330, 45%). Half of the donations were collected around London, including Edgware, Tooting and West End Donor Centres (165/330). Social deprivation scores were calculated based on postcode and Acorn classification [14]: most donors were affluent achievers, classed as the financially most successful people in the UK based on this model (120/330; 36%). Hospitalisation data retrieved from NHS Digital and via an internet form completed by donors at registration demonstrated that only 10% of the donors had been hospitalised with COVID-19 (33/330).

**Figure 1:**
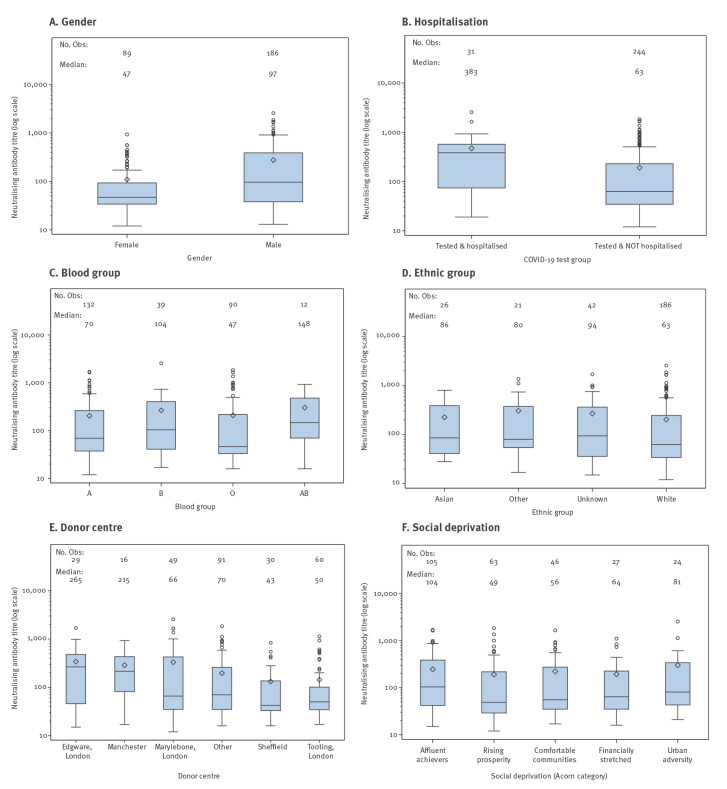
Median neutralising antibody levels against SARS-CoV-2, by blood donor characteristics, England, 22 April–12 May 2020 (n = 275)

COVID-19: coronavirus disease; SARS-CoV-2: severe acute respiratory syndrome coronavirus 2.

**Figure 2:**
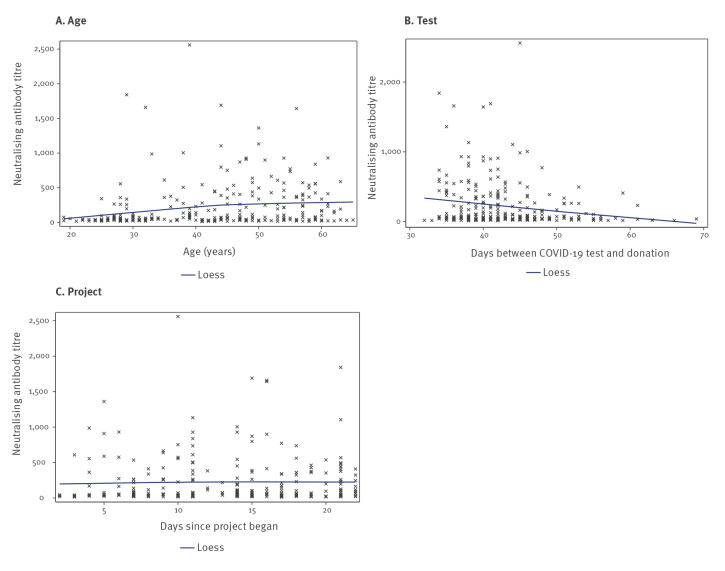
Neutralising antibody levels against SARS-CoV-2, by age, days post diagnosis and day of donation, England, 22 April–12 May 2020 (n = 275)

COVID-19: coronavirus disease; SARS-CoV-2: severe acute respiratory syndrome coronavirus 2.

**Figure 3:**
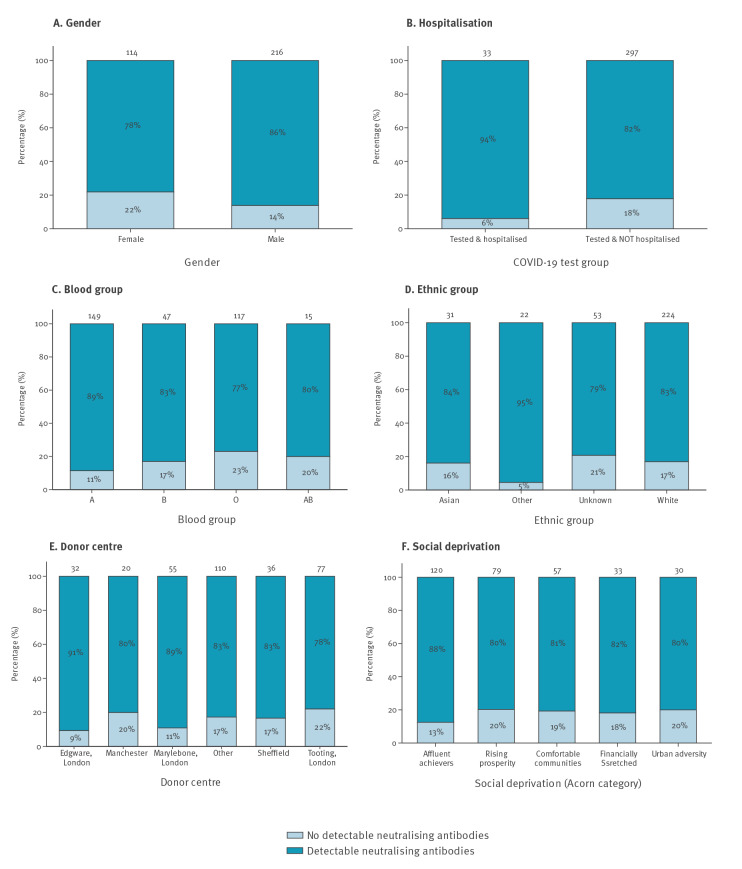
Proportion of donors with or without neutralising antibodies against SARS-CoV-2, by blood donor characteristics, England, 22 April–12 May 2020 (n = 330)

COVID-19: coronavirus disease; SARS-CoV-2: severe acute respiratory syndrome coronavirus 2.

**Figure 4:**
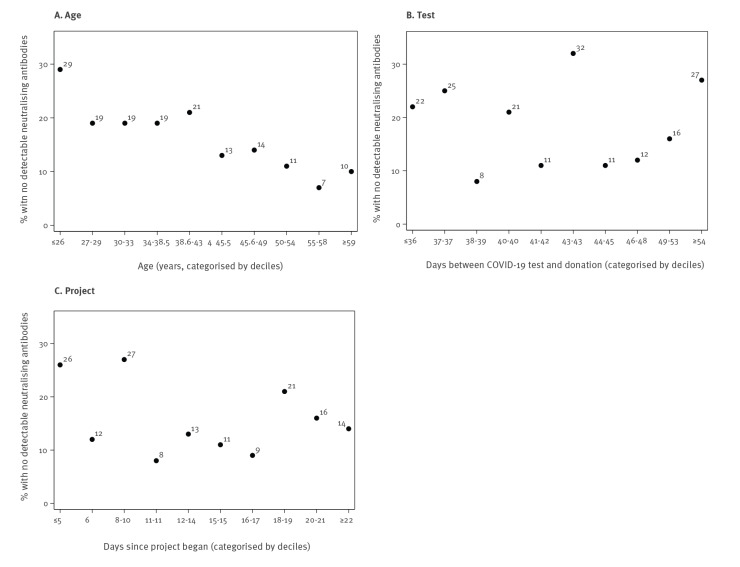
The proportion of blood donors without detectable neutralising antibodies against SARS-CoV-2, by age, days post diagnosis and day of donation, England, 22 April–12 May 2020 (n = 330)

COVID-19: coronavirus disease; SARS-CoV-2: severe acute respiratory syndrome coronavirus 2.

Among the 330 donors, 275 had detectable neutralising antibodies against SARS-CoV-2 (titre range: 1:12–1:2,560; median titre: 1:69; interquartile range 1:35–1:280). For these 275 donors, median levels of neutralising antibodies were higher in men compared with women (1:97 vs 1:47), in those hospitalised compared with non-hospitalised (1:383 vs 1:63), in those with blood group AB compared with other groups (1:148 vs 1:104 for group B, 1:70 for group A and 1:47 for group O; however, the number of donors with AB blood group was small: n = 12) and in those who donated at Edgware Donor centre (1:265 vs 1:215 in Manchester, 1:66 in the West End Donor centre, 1:50 in Tooting, 1:43 in Sheffield and 1:70 in other areas; Figures 1 and 2; Supplementary Table S1). Donors in the white ethnicity group had lower levels of neutralising antibodies than other ethnic groups (1:63 vs 1:86 (Asian), 1:80 (other) and 1:94 (unknown)). Similar neutralising antibody levels were observed between different social deprivation groups. We observed that neutralising antibody levels increased with increasing age, whereas antibody levels decreased with increasing time interval between SARS-CoV-2 diagnosis and donation. However, neutralising antibody levels observed for donations collected daily remained similar during the study period suggesting that the time of donation (i.e. whether they donated in the beginning or at the end of the study period; everybody donated only once) did not influence the neutralising antibody titres. In general, the factors associated with lower antibody levels (Figures 1 and 2) were also associated with likelihood of donors not having any detectable antibodies (Figures 3 and 4). For example, 22% of female donors had no detectable neutralising antibodies compared with 14% of male donors.

## Association between host factors and neutralising antibody levels

We further assessed these factors in two different multivariable analyses. For both models, we used a stepwise variable selection method where donor variables were retained in the model if they reduced the model deviance significantly (p < 0.1) according to the likelihood ratio test. Interactions between the variables could not be considered because of the small number of donors in this study.

Firstly, we identified factors associated with the probability of not having detectable neutralising antibodies in 313 donors. Two donors were excluded because blood group data were missing and 15 donors with AB blood group were excluded as the model was highly sensitive to this group because of small numbers. Therefore, the results of this model could not be generalised for AB donors. We set up an indicator variable to indicate whether neutralising antibody level was recorded as negative and developed a multivariable logistic regression model to assess the probability of convalescent plasma donors not having measurable neutralising antibodies. 

Hospitalisation was confounded with blood group; it was significant (p = 0.07) only when blood group was not included in the model. Blood group was significant irrespective of whether the hospitalisation was in the model. Blood group A donors had significantly lower odds of not having detectable neutralising antibodies than group B and O donors. The percentage hospitalised by blood group illustrates how this information is confounded with the hospitalisation term (interpretation for group AB donors is limited owing to small numbers): Blood group A (21 hospitalised of 149 donors; 14%), blood group B (4/47; 9%), blood group O (4/117; 3%), blood group AB (4/15; 27%). The term ‘previous blood donor’ was sensitive to influential values. Excluding the strongest influential values from the analysis just led to other observations becoming influential and therefore, all observations were included. However, the parameter estimates and hence magnitude of the OR should be interpreted with caution. Based on this model, younger age, female gender, blood group O and not being a previous blood donor were associated with non-detectable neutralising antibody response (Table 1).

**Table 1:** Logistic regression model for non-detection of neutralising antibodies, England, 22 April–12 May 2020 (n = 313)

Factor	Categorisation	OR	95% CI	p value
Age	Linear variable^a^	0.96	0.93–0.99	0.002
Hospitalised	Yes	Not done	Not done	0.25
	No			
Ethnic group	Asian	Not done	Not done	0.36
	Other			
	Unknown			
	White			
Gender	Female	1	Reference	0.009
	Male	0.41	0.21–0.80	
Donor centre	Edgware	Not done	Not done	0.56
	Manchester			
	Sheffield			
	Tooting			
	West End Donor Centre			
	Other			
Blood group	A	0.34	0.17–0.70	0.01
	B	0.64	0.26–1.58	
	O	1	Reference	
	AB	Excluded	Excluded	
Previous blood donor	Yes	0.36	0.11–1.16	0.06
	No	1	Reference	
Social deprivation indicator^b^	Affluent achievers	Not done	Not done	0.98
	Rising prosperity			
	Comfortable communities			
	Financially stretched			
	Urban adversity			
Days since project began	Linear variable	Not done	Not done	0.42
Days since diagnosis	Linear variable^c^	Not done	Not done	0.97

CI: confidence interval; OR: odds ratio.

^a^ A nonlinear term for age was tested but found to be non-significant (p = 0.9).

^b^ Social deprivation scores were calculated based on postcode and Acorn classification [14], which divides the population into five categories. Based on that model, ‘affluent achievers’ are the most financially successful and healthy people in the United Kingdom, ‘rising prosperity’ includes generally younger, well educated, and mostly prosperous people living in our major towns and cities, ‘comfortable communities’ are the middle graders in terms of social and financial wellbeing, whereas the ‘financially stretched’ group includes mostly people with modest lifestyles and less than average income and the ‘urban adversity’ group includes people who are experiencing the most difficult social and financial conditions.

^c^ A nonlinear term for ‘days since diagnosis’ was tested but found to be non-significant (p = 0.26).

Donors with detectable neutralising antibodies were selected for the second model (n = 275, but a further 10 donors were excluded because data on social deprivation were missing). We used a multivariable gamma generalised linear model (GLM) to identify the factors associated with neutralising antibody titres. All variables in the model, except for gender, suffered from influential values. The two highest neutralising antibody titres measured (1:2,560 and 1:1,841) were the most influential but excluding these values (i) would be excluding genuine observations and hence artificially reduce the sample size just to improve model fit and (ii) was found to cause other values to become influential values. The value of these parameter estimates should therefore not be interpreted, only their direction and significance. This analysis demonstrated a significant association between increasing neutralising antibody titres and increasing age, hospitalisation, male gender, donor centre (highest titres in Edgware) and donating sooner after SARS-CoV-2 diagnosis (Table 2). Social deprivation (whereby donors from the most deprived areas in large cities and towns in England had higher antibody levels) and timing (whereby those who donated later in the study period had higher antibody levels) were also significant factors.

**Table 2:** Gamma GLM model for neutralising antibody levels, England, 22 April–12 May 2020 (n = 265)

Factor	Categorisation	Exp (par. est)	95% CI	p value
Age	Linear variable^a^	1.02	1.01–1.03	0.0001
Hospitalisation	Yes	2.25	1.52–3.32	p < 0.0001
	No	1	Reference	
Ethnic group	Asian	Not done	Not done	0.64
	Other			
	Unknown			
	White			
Gender	Female	1	Reference	p < 0.0001
	Male	2.41	1.86–3.14	
Donor centre	Edgware	2.87	1.81–4.54	p < 0.0001
	Manchester	2.35	1.32–4.19	
	Sheffield	0.81	0.50–1.33	
	Tooting	1	Reference	
	West End Donor Centre	1.99	1.38–2.87	
	Other	1.26	0.88–1.80	
Blood group	A	Not done	Not done	0.81
	B			
	O			
	AB			
Previous blood donor	Yes	Not done	Not done	0.30
	No			
Social deprivation indicator^b^	Affluent achievers	0.65	0.42–1.02	0.06
	Rising prosperity	0.55	0.35–0.88	
	Comfortable communities	0.60	0.37–0.98	
	Financially stretched	0.47	0.27–0.82	
	Urban adversity	1	Reference	
Days since project began	Linear variable	1.04	1.02–1.07	0.001
Days since diagnosis^c^	Linear variable	0.94	0.92–0.96	p < 0.0001

CI: confidence interval; Exp (par. est): the exponentiated parameter estimates; GLM: generalised linear model.

^a^ A nonlinear term for age was tested but found to be non-significant (p = 0.25).

^b^ Social deprivation scores were calculated based on postcode and Acorn classification [14], which divides the population into five categories. Based on that model, ‘affluent achievers’ are the most financially successful and healthy people in the United Kingdom, ‘rising prosperity’ includes generally younger, well educated, and mostly prosperous people living in our major towns and cities, ‘comfortable communities’ are the middle graders in terms of social and financial wellbeing, whereas the ‘financially stretched’ group includes mostly people with modest lifestyles and less than average income and the ‘urban adversity’ group includes people who are experiencing the most difficult social and financial conditions.

^c^ A nonlinear term for ‘days since diagnosis’ was tested but found to be non-significant (p = 0.91).

## Discussion

Although we used two different models testing different outcomes, several factors shared associations with neutralising antibodies in both models. It is clear that women and younger donors were more likely not to have measurable neutralising antibodies, and among those with detectable neutralising antibodies, the levels were higher in men and in older donors. Higher neutralising antibody levels were also seen in those who had been hospitalised and in those who donated in Edgware or Manchester; however, donor centre was not significant in determining whether or not a donor lacked neutralising antibodies. Interestingly, neutralising antibody levels decreased as the time between SARS-CoV-2 diagnosis and donation increased. 

It is difficult to explain the differences between donations given in different parts of England without further analysis of ethnicity and other possible factors associated with SARS-CoV-2 infection. Interestingly, ethnicity was not found to be a significant factor in the multivariable models, whereas donor centre was a significant factor only in the gamma GLM. The models might suffer from confounding and multicollinearity. Furthermore, social deprivation score remained an independent factor in the gamma GLM. It is noticeable that ethnicity data were not available or remained uncategorised for 23% of donors and may therefore have influenced the data analysis.

Interestingly, blood group O and not being a previous blood donor were both associated with non-detectable antibody response in our study. However, we cannot say based on these data whether the latter association was causal or not, but it could reflect a testing seeking behaviour attracting new blood donors during the early stages of pandemic. Furthermore, in keeping with our findings here, neutralising antibodies levels were lower in French blood donors with blood group O than in other groups [15], whereas based on early epidemiological evidence, women with blood group A were more susceptible to SARS-CoV-2 infection [16]. These observations have also been confirmed in a large genome-wide association study where a higher risk of infection was seen in those with blood group A than in any other group and a protective effect of blood group O on SARS-CoV-2 infection was demonstrated [17].

Our findings are consistent with data on 126 convalescent donors in the United States, demonstrating a similar association between male sex, older age, SARS-CoV-2 infection requiring a hospitalisation, and higher neutralising antibody titres [10,18]. Although it has been proposed that higher antibody levels in male and older patients simply relate to COVID-19 severity [19], our model proposes that they remain associated with higher neutralising antibody titre levels after adjusting for hospitalisation. Although it is unclear what factors are governing these sex- and age-related antibody responses, this information can be used for targeted donor recruitment for convalescent plasma.

Both models suffered from influential values limiting interpretation of the results. In particular, the gamma GLM should only be used to infer significance of variables and the direction of each parameter estimate; parameter estimates should not be interpreted. Odds ratios from the logistic regression model should also be interpreted with caution. Both models would benefit greatly from a larger dataset. Furthermore, our data on social deprivation should be considered with caution as we have not evaluated how well the Acorn classification, largely focusing on financial aspects, reflects the social deprivation associated with health outcomes.

## Conclusions

Until effective antiviral treatments and vaccines against the COVID-19 pandemic become available, convalescent plasma therapy is an existing option that can be used against this infection. It is important that the convalescent plasma contains high titres of neutralising antibody as the use of low-titre plasma can prevent or prolong evaluation of its efficacy in clinical trials. Older male donors with a previous SARS-CoV-2 infection leading to hospitalisation were in our study the most likely to have high neutralising antibody titres. This knowledge can support fast and practical recruitment strategies so that limited testing resources can be targeted to those most likely to harbour therapeutic levels of neutralising antibodies. It can also inform selection of places and time periods when resources for antibody testing are limited.

## Supplementary Data

528000 Supplement

## References

[1] Huang C Wang Y Li X Ren L Zhao J Hu Y Clinical features of patients infected with 2019 novel coronavirus in Wuhan, China. Lancet. 2020;395(10223):497-506. 10.1016/S0140-6736(20)30183-5 31986264 PMC7159299

[2] Wang Y Zhang D Du G Du R Zhao J Jin Y Remdesivir in adults with severe COVID-19: a randomised, double-blind, placebo-controlled, multicentre trial. Lancet. 2020;395(10236):1569-78. 10.1016/S0140-6736(20)31022-9 32423584 PMC7190303

[3] Folegatti PM Ewer KJ Aley PK Angus B Becker S Belij-Rammerstorfer S Safety and immunogenicity of the ChAdOx1 nCoV-19 vaccine against SARS-CoV-2: a preliminary report of a phase 1/2, single-blind, randomised controlled trial. Lancet. 2020;396(10249):467-78. 10.1016/S0140-6736(20)31604-4 32702298 PMC7445431

[4] Duan K Liu B Li C Zhang H Yu T Qu J Effectiveness of convalescent plasma therapy in severe COVID-19 patients. Proc Natl Acad Sci USA. 2020;117(17):9490-6. 10.1073/pnas.2004168117 32253318 PMC7196837

[5] Shen C Wang Z Zhao F Yang Y Li J Yuan J Treatment of 5 critically ill patients with COVID-19 with convalescent plasma. JAMA. 2020;323(16):1582-9. 10.1001/jama.2020.4783 32219428 PMC7101507

[6] Zhang L Pang R Xue X Bao J Ye S Dai Y Anti-SARS-CoV-2 virus antibody levels in convalescent plasma of six donors who have recovered from COVID-19. Aging (Albany NY). 2020;12(8):6536-42. 10.18632/aging.103102 32320384 PMC7202482

[7] Ibrahim D, Dulipsingh L, Zapatka L, Eadie R, Crowell R, Williams K, et al. Factors associated with good patient outcomes following convalescent plasma in COVID-19: A prospective phase II clinical trial. medRxiv 2020. Available from: https://doi.org/10.1101/2020.08.27.20183293 PMC7502154 32951151

[8] Li L Zhang W Hu Y Tong X Zheng S Yang J Effect of convalescent plasma therapy on time to clinical improvement in patients with severe and life-threatening COVID-19: a randomized clinical trial. JAMA. 2020;324(5):460-70. 10.1001/jama.2020.10044 32492084 PMC7270883

[9] Avendano-Sola C, Ramos-Martinez A, Munez-Rubio E, Ruiz-Antoran B, Malo de Molina R, Torres F, et al. Convalescent plasma for COVID-19: A multicenter, randomized clinical trial. medRxiv 2020. Available from: https://doi.org/10.1101/2020.08.26.20182444v3

[10] Liu STH Lin HM Baine I Wajnberg A Gumprecht JP Rahman F Convalescent plasma treatment of severe COVID-19: a propensity score-matched control study. Nat Med. 2020. 10.1038/s41591-020-1088-9 32934372

[11] Joyner M, Senefeld JW, Klassen SA, Mills JR, Johnson PW, Theel ES, et al. Effect of convalescent plasma on mortality among hospitalized patients with COVID-19: initial three-month experience. medRxiv 2020. Available from: https://doi.org/10.1101/2020.08.12.20169359

[12] Harvala H Mehew J Robb ML Ijaz S Dicks S Patel M Convalescent plasma treatment for SARS-CoV-2 infection: analysis of the first 436 donors in England, 22 April to 12 May 2020. Euro Surveill. 2020;25(28):2001260. 10.2807/1560-7917.ES.2020.25.28.2001260 32700670 PMC7376844

[13] Harvala H, Robb ML, Watkins N, Ijaz S, Dicks S, Patel M, et al. Convalescent plasma therapy for the treatment of patients with COVID-19: Assessment of methods available for antibody detection and their correlation with neutralising antibody levels. medRxiv 2020. Available from: https://doi.org/10.1101/2020.05.20.20091694v1 PMC8246874 33333627

[14] Consolidated Analysis Centers, Inc. (CACI). ACORN categorisations of deprivation in the UK based on geodemographic segmentation of the UK’s population. London: CACI; 2014. Available from: https://acorn.caci.co.uk/downloads/Acorn-User-guide.pdf

[15] Gallian P Pastorino B Morel P Chiaroni J Ninove L de Lamballerie X Lower prevalence of antibodies neutralizing SARS-CoV-2 in group O French blood donors. Antiviral Res. 2020;181:104880. 10.1016/j.antiviral.2020.104880 32679056 PMC7362788

[16] Fan Q Zhang W Li B Li DJ Zhang J Zhao F Association between ABO blood group system and COVID-19 susceptibility in Wuhan. Front Cell Infect Microbiol. 2020;10:404. 10.3389/fcimb.2020.00404 32793517 PMC7385064

[17] Ellinghaus D Degenhardt F Bujanda L Buti M Albillos A Invernizzi P Genomewide association study of severe COVID-19 with respiratory failure. N Engl J Med. 2020;383(16):1522-34. 10.1056/NEJMoa2020283 32558485 PMC7315890

[18] Klein S, Pekosz A, Park HS, Ursin R, Shapiro J, Benner S, et al. Sex, age, and hospitalization drive antibody responses in a COVID-19 convalescent plasma donor population. medRxiv 2020. Available from: https://www.medrxiv.org/content/10.1101/2020.06.26.20139063v1 10.1172/JCI142004 PMC7598041 32764200

[19] Scully EP Haverfield J Ursin RL Tannenbaum C Klein SL Considering how biological sex impacts immune responses and COVID-19 outcomes. Nat Rev Immunol. 2020;20(7):442-7. 10.1038/s41577-020-0348-8 32528136 PMC7288618

