# Supplementary material for: Convalescent plasma for COVID-19: male gender, older age and hospitalisation associated with high neutralising antibody levels, England, 22 April to 12 May 2020
Source: Euro Surveill. 2020 Nov 12;25(45):2001754. doi: 10.2807/1560-7917.ES.2020.25.45.2001754 (PMC7667632; doi:10.2807/1560-7917.ES.2020.25.45.2001754)
Supplement: Supplement [file 20-01754_Supplement_Harvala.pdf]

Supplementary Table 1. Convalescent donor characteristics and other factors investigated in this study (n=330 for all donors; n=275 for donors with neutralising antibodies).

This supplementary material is hosted by Eurosurveillance as supporting information alongside the article "Convalescent plasma for COVID-19: Male gender, older age and hospitalisation associated with high neutralising antibody levels, England, 22 April to 12 May 2020" on behalf of the authors, who remain responsible for the accuracy and appropriateness of the content. The same standards for ethics, copyright, attributions and permissions as for the article apply. Supplements are not edited by Eurosurveillance and the journal is not responsible for the maintenance of any links or email addresses provided therein.

	Total numbers (n=330)	Number with measurable neutralising antibodies (n=275)
**Gender**		
Female	114	89
Male	216	186
**Hospitalisation**		
Yes	33	31
No	297	244
**Blood group**		
A	149	132
B	47	39
O	117	90
AB	15	12
Not known	2	2
**Ethnic group**		
Asian	31	26
Other	22	21
Unknown	53	42
White	224	186
**Donor centre**		
Edware	32	29
Manshester	20	16
Marylebone	55	49
Other	110	91
Sheffied	36	30
Tooting	77	60
**Social deprivation**		
Affluent achievers	120	105
Rising prosperity	79	63
Comfortable communities	57	46
Financially stretched	33	27
Urban adversity	30	24
Not known	11	10
